# Role of CC-chemokine ligand 2 in gynecological cancer

**DOI:** 10.1186/s12935-022-02763-z

**Published:** 2022-11-19

**Authors:** Jia-Jia Zhang, Wei Liu, Guo-Zhen Xing, Li Xiang, Wen-Ming Zheng, Zhen-Ling Ma

**Affiliations:** 1 College of Life Sciences, Henan Agricultural University, Zhengzhou, 450002 China; 2 Henan Provincial People’s Hospital/People’s Hospital of Zhengzhou University, 7 Weiwu Road, Jinshui District, Zhengzhou, 450000 China

**Keywords:** CCL2, CCL2/CCR2 axis, Breast cancer, Ovarian cancer, Cervical cancer

## Abstract

Gynecological cancer is one of the most severe diseases that threaten the lives and health of women worldwide. Its incidence rate increases with each passing year and becomes more prevalent among young people. The prognosis of gynecological cancer remains poor despite significant advances in surgical removal and systemic chemotherapy. Several chemokines play a role in the progression of gynecologic cancers. CCL2 (CC-chemokine ligand 2), also termed MCP-1 (monocyte chemotactic protein 1), plays a significant physiological role in monocyte cell migration and the inflammatory response. Recent studies have demonstrated that CCL2 plays a pro-tumorigenic function in the tumor microenvironment. According to previous studies, CCL2 plays a significant role in the occurrence and development of gynecological cancers. Furthermore, recent studies noted that CCL2 could be a potential diagnostic biomarker and prognostic predictor. The purpose of this paper is to review the role of CCL2 in the occurrence and development of gynecological cancers and to discuss the potential therapeutic strategy of CCL2 for gynecological cancers, with a primary focus on breast cancer, ovarian cancer, cervical cancer, and endometrial cancer.

## Introduction

Gynecological cancer is one of the most prevalent diseases in women worldwide. There has been an increase in the incidence of gynecological cancer in recent years, and the affected population is younger. Early gynecological malignant tumors respond well to traditional treatments. However, for advanced tumors, although surgery and chemotherapy can remove or reduce tumor tissue, in most cases, it is difficult to effectively deal with metastasis, recurrence, and a poor prognosis. Chemokines, which are small heparin-binding proteins, play an important role in inflammation, immune response, and cancer development. Chemokines can be classified into four subfamilies depending on the position of the conserved cysteine residue, namely CXC, CC, CX_3_C, and C. Certain chemokines attract monocytes to definite sites throughout the body [1]. Chemokines play a dual role in tumor development. In the tumor microenvironment, several chemokines promote tumor progression and angiogenesis by recruiting tumor-associated macrophages. Other chemokines function as antitumor substances by inhibiting angiogenesis, allowing immune cells to respond to tumors, and killing cancer cells [2]. CC-chemokine ligand 2 (CCL2) is a member of the CC chemokine subfamily. It is highly expressed in different cancers and is associated with tumorigenesis and metastasis of prostate, bladder, colorectal, and breast cancer [3–5]. Furthermore, the expression of CCL2 is elevated in breast, ovarian, and cervical cancer, indicating the pivotal role of CCL2 in gynecological malignancies. In this review, we summarize the evidence for the involvement and potential therapeutic target of CCL2 in gynecological malignancies, focusing on breast, ovarian, cervical, and endometrial cancers.

## Biological characteristics of CCL2

**Fig. 1:**
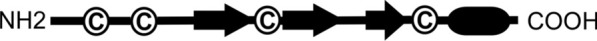
Structure of CCL2. C shows cysteine residues. The bold arrow shows β-plated. The cylinder shows α-helical structure

Chemokine (C-C motif) ligand 2 (CCL2) is the first CC chemokine discovered in humans, which belongs to the CC subfamily. CCL2 has three discrete domains: adjacent cysteines close to the N-terminal, three antiparallel β-pleated sheets, and an α-helical structure (Fig. 1). *CCL2* is positioned on chromosome 17 (chr.17, q11.2). A human CCL2 molecule is 13 kDa in size and consists of 76 amino acids. Because it had previously been discovered in tumor cells in vitro, CCL2 was originally called a tumor-derived chemokine [6]. CCL2 is expressed and secreted by a variety of cell types, including epithelial cells, smooth muscle cells, fibroblasts, epithelial cells, and T cells [7]. CCL2 is a multifunctional factor that chemotactically attracts monocytes and immune cells, such as T cells and natural killer cells, to specific sites [8–10]. It is also a double-edged sword in the incidence and development of diseases [11]. As part of the wound healing process, CCL2 promotes the formation of new blood vessels at the wound site [12, 13]. On the other hand, CCL2 stimulates tumor proliferation, migration, invasion, and angiogenesis and inhibits the autoimmune system [14–19]. Furthermore, previous studies suggest that the expression of CCL2 has a strong relationship with cancer prognosis [20, 21].

## The CCL2-CCR2 signaling axis

**Fig. 2:**
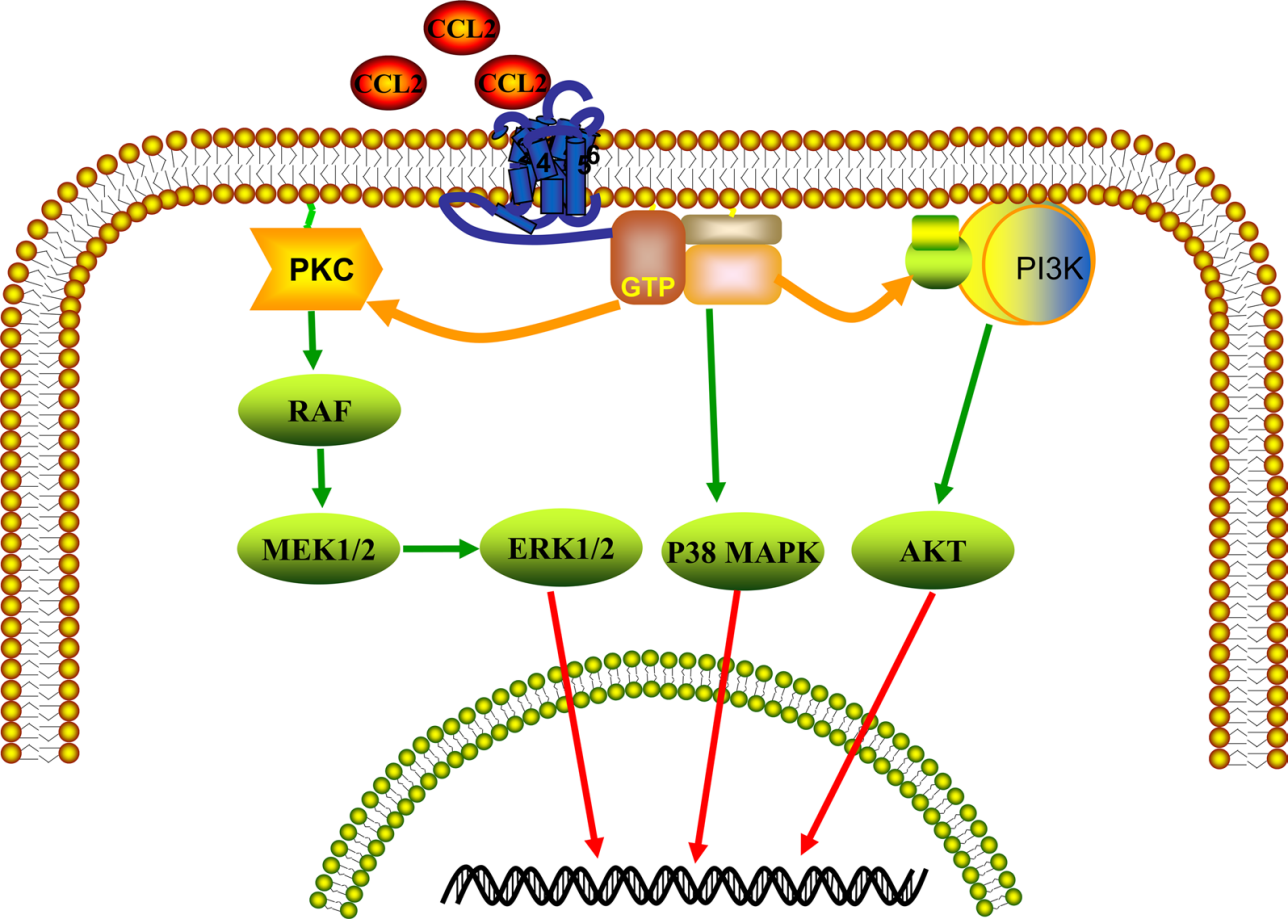
The diagram of the CCL2-CCR2 axis in cellular reactions

Chemokine receptors are recognized as G-protein-coupled receptors (GPCRs). GPCRs are seven-transmembrane receptors and are expressed on numerous cells. The activated GPCRs are responsible for the formation of cAMP, DAG, and IP3 and protein kinase A and C activation. Although CCL2’ receptors include CCR2 and CCR4, CCL2 primarily mediates its functions through binding to CCR2 and triggering a series of signal transduction reactions [22]. CCR2 is expressed on the surface of multiple cell types, monocytes, dendritic cells, immune cells, natural killer cells, and tumor cells. As a CCR2-binding protein, CCL2 activates intracellular pathways, including phosphatidylinositol-3-OH kinase (PI3K), mitogen-activated protein kinases (MAPK), and protein kinase C (Fig. 2). Hence, the CCL2-CCR2 signaling axis plays a key role in several physiological processes, including disease progression.

The CCL2-CCR2 signaling axis has been shown to play an important role in cancer progression. The first and most important line of immune defense in the central nervous system (CNS) is microglia, which are the brain and spinal cord’s counterparts of macrophages (CNS). Most glioma cells express CCL2. Due to CCR2 expression, Glioma cells are surrounded by microglia. According to Zhang et al., CCL2 expressed by glioma cells stimulates microglia to produce IL-6, and IL-6 produced by microglia, in turn, promotes the invasiveness of glioma cells. These results suggest that the CCL2/CCR2/IL-6 axis could be a potential therapeutic target for glioma [23]. The inhibition of the CCL2-CCR2 axis may result in an antitumor function in a malignant glioma model [24]. Estrogen up-regulates CCL2 expression in ER^+^ breast cancer cells through the Twist/PI3K/AKT/NF-κB signaling pathway. ER^+^ breast cancer cells proliferate, migrate, and invade, in part due to up-regulated CCL2 interacting with its receptor, CCR2 [25]. Coincidentally, Yao et al. also discovered that CCL2 increases cell proliferation and cell cycle progression in MCF breast cancer cells [26]. Cell proliferation and trafficking are controlled by the CCL2-CCR2 signaling pathway in acute myeloid leukemia [27].

Considering the chemotactic effect of CCL2 on monocytes, recent research has found that CCL2 is involved in the cross-talk between tumor cells and tumor-associated macrophages (TAMs) in the tumor microenvironment. Tumor-derived CCL2, in combination with CCR2 on the surface of monocytes, recruits monocytes to infiltrate the tumor microenvironment or metastatic locations and promotes the TAMs to polarize into M2-TAMs [28, 29]. TAMs accumulation was eliminated in esophageal squamous cell carcinoma when the CCL2-CCR2 axis was blocked, and TAMs-induced tumor cell evasion was suppressed via the PD-1 signaling pathway [30]. Furthermore, CCL2 secreted by TAMs targets tumor cells, directly promotes proliferation and metastasis and mediates drug resistance in tumor cells [31]. Using a mouse model with endothelial-specific deletion of CCR2, Roblek et al. demonstrated that CCL2-CCR2 signaling is necessary for tumor cell extravasation and lung metastases [32]. With further research, it has been elucidated that the CCL2-CCR2 axis is involved in the occurrence and development of more and more diseases, while its mechanisms in diseases have not been clarified. As a result, elucidation mechanisms of the CCL2-CCR2 axis in diseases will provide a broader prospect for diagnosing and treating.

## Role and underlying mechanisms of CCL2 in gynecological cancer

### CCL2 and breast cancer

Breast cancer is a malignant tumor that develops when mammary epithelial cells proliferate uncontrollably. It is one of the most dangerous cancers in the world that threatens the life and health of women, and it is also a major risk factor for other cancer-related diseases [33]. Surgical excision and systemic chemotherapy are still the most common treatments for breast cancer, but enormous damage is done to women’s bodies, and the problem of resistance to chemotherapy still exists. The focus of breast cancer prevention and treatment has shifted to finding more effective treatment approaches. CCL2 has been found to play a crucial role in the growth, metastasis, and invasion of breast cancer cells in recent research (Table 1). As a result, studying the function of CCL2 in the progression of breast cancer can provide new ideas for the screening, diagnosis, and treatment of breast cancer. CCL2 is a diagnostic marker that can be used to predict the early recurrence of breast cancer. The high expression of CCL2 in the plasma of breast cancer patients is confirmed by enzyme-linked immunosorbent assay (ELISA), but the low concentration of CCR2. The detection of plasma CCL2 and CCR2 combined with CA 15-1 can be used as a biomarker for breast cancer diagnosis [34]. Furthermore, studies have shown that CCL2 is highly expressed in tumor epithelial cells, and many CD14-positive cells are infiltrated in primary tumors, implying that high levels of CCL2 and CD14-positive cells can serve as diagnostic markers for early recurrence of breast cancer [35]. CCL2 is highly expressed in numerous breast cancer lines as well as the monocytes and stroma cells in the tumor microenvironment. Furthermore, CCL2 expression is higher in metastatic breast tumors compared to primary breast tumors. These findings imply that a high level of CCL2 may be involved in the migration of breast cancer cells [36]. Studies have revealed that CCL2 improves the survival, motion ability, and migration of mammary carcinoma cell lines 4T1, MCF-7, PyVmT, and MDA-MB-231 by activating phosphorylation of Smad3 and p42/44 MAPK [37]. Meanwhile, CCL2 overexpression stimulated the proliferation of breast cancer cell line 4T1 [38]. Furthermore, CCL2 plays an important function in the drug-resistant of breast cancer cells. Li et al. explained that tumor-associated macrophages secrete CCL2 and CCL2 promote resistance to tamoxifen through PI3K/Akt/mTOR signaling pathway [31].

**Table 1:** Expression and function of CCL2 in gynecological cancer

Cancer types	High or low expression	Pro(+) or Anti(−) tumor	Prognosis	References
Breast cancer	High	(+)	Early recurrence	[34, 35, 39–42]
Ovarian cancer	High	(+)	High histological grade	[47, 49, 51–54]
Cervical cancer	Low or high	(+)	Poor prognosis and overall survival	[57, 58, 60, 61]
Endometrial cancer	High	(+)	Accelerate tumor progress and reduce survival	[62, 66–68]

CCL2, as a chemotactic cytokine, can recruit immune monocytes to promote tumor progression. A mouse model of MMTV-HER2 infection demonstrated that tumor cells and other myeloid cells from early breast lesions produced CCL2, which attracted macrophages to the site of early breast lesions. Secretion of Wnt-1 by intra-epithelial macrophages induced reduction in E-cadherin connectivity, thus, promoting early spread and metastasis of breast cancer [39]. CCL2 can also attract inflammatory monocytes to the pulmonary metastasis focuses, allowing breast cancer cells to spread to lung metastases [40]. CCL2 binds to CCR2 on the surface of metastasis-associated macrophages, causing the macrophages to generate CCL3 and increasing breast cancer lung metastasis [41]. CCL2 encouraged macrophages to produce CXCL12 through synergistic interaction with IL-1, which encouraged angiogenesis [42].

In a recent study, Fang et al. proficiently reduced the CCL2 expression by using a novel gene silencing method Ca-TAT/siRNA. Their results indicated that CCL2 silencing inhibited triple negative tumor cell growth and metastasis by decreasing cancer stem cell renewal and M2 macrophage recruitment [43]. Breast cancer can be treated with CCL2 neutralizing antibodies and the same outcomes were observed by targeting CCR2. Brummer et al. showed that selective targeting of CCR2 in the mammary epithelium repressed breast tumor growth, invasion, and decreased angiogenesis [44]. Blocking CCL2 has been reported to lead to excessive metastasis and accelerated death. Therefore, inhibition of CCL2 or CCL2/CCR2 may be a potential approach to the treatment of breast cancer. An additional study reported that continued administration of CCL2-neutralizing antibodies for more than four weeks leads to little therapeutic effect, with CCL2 levels increasing over time [45]. As a result, CCL2 and CCL2/CCR2 as therapeutic targets for breast cancer remain to be further investigated.

### CCL2 and ovarian cancer

Ovarian cancer is a malignant tumor on the ovaries. It comprises epithelial ovarian cancer, germ ovarian cancer, specific sex cord-stromal ovarian cancer, and metastatic ovarian cancer, of which epithelial ovarian cancer is the most common. Due to a lack of early symptoms and limited screening and diagnosis methods, ovarian cancer is often diagnosed in late stages, resulting in poor treatment outcomes [46]. Ovarian cancer has a lower incidence than other gynecological tumors, but it has a high mortality rate and poses a serious threat to women’s health. Serum CCL2 levels in patients with primary ovarian cancer are significantly higher than those in women with benign ovarian cysts and healthy women, according to Hefler et al. [47]. On the other hand, another study found that CCL2 is up-regulated in MA-148 ovarian cancer cells when treated with paclitaxel and/or carboplatin [48]. Exogenous CCL2 stimulated the migration and adhesion of SKOV-3 cells, which can be reduced by adding a CCR2 antagonist [49]. Recently, our group purified human recombinant CCL2 proteins and showed that exogenous CCL2 improved ovarian cancer cell proliferation by stimulating the MAPK/ERK signaling pathway and regulating JUN, RELB, and NF-κB2 expression levels [50]. CCL2 also stimulates the progression of ovarian cancer by enhancing angiogenesis. Deng et al. found that recombinant CCL2 down-regulated TNFSF15 expression, which is a negative regulator of neovascularization, thus promoting tumor angiogenesis and accelerating ovarian cancer expansion [51]. These results suggest that CCL2 may serve as a potential therapeutic target for the treatment of ovarian cancer.

CCL2 has also been shown to play a role in the action of other cells on ovarian cancer cells. CCL2 from mesenchymal stromal cells act on ovarian cancer cells and induces IL-6 secretion for chemoresistance dependent on IL-6 and PYK2 [52]. Cancer-associated mesothelial cells play a key role in the peritoneal metastasis of ovarian cancer. They enhance the invasion of epithelial ovarian cancer cells by generating CCL2 through the P38-MAPK pathway. In addition, inhibition of CCL2 by neutralizing antibodies reduced the invasion of epithelial ovarian cancer cells [53]. CCL2 is also involved in the communication between other stromal cells and ovarian cancer cells. Omental adipocytes expressed a high level of CCL2 in an experimental mouse model of ovarian cancer peritoneal metastasis. Binding to its cognate receptor CCR2, CCL2 expedited the migration and invasion of ovarian cancer cells and improved cisplatin resistance. The enhanced metastasis and invasion of ovarian cancer cells can be eliminated by blocking CCL2/CCR2 signaling transduction with CCL2 neutralizing antibodies or a CCL2 gene knockout defect [54]. These studies suggest that CCL2/CCR2 axis might be a novel target for ovarian cancer therapy that could benefit patients with the disease.

### CCL2 and cervical cancer

Human papillomavirus (HPV) infection is the most common cause of cervical cancer in women. According to statistics, approximately 570,000 new cases of cervical cancer were diagnosed in 2018, and approximately 311,000 deaths [55]. Furthermore, China accounted for about a third of all new cases worldwide. However, relapses in patients continue to be associated with a high mortality rate despite significant advances in early detection, vaccination prevention, and treatment [56]. Studies have shown that CCL2 is present in cervical cancer cells and their precancerous lesions. CCL2 is greatly expressed in the tumor microenvironment of cervical cancer. Tumor cells express less CCL2, while other cells express more. Human papillomavirus E6/E7 oncoproteins may play a role in this process. Kleine et al. have shown that E6 and E7 oncoproteins selectively inhibit CCL2 expression in cervical epithelial and epidermal cells. Furthermore, Riethdorf S et al. demonstrated that E6/E7 oncogenes negatively regulate CCL2 transcription [57, 58]. Although cervical cancer cells produce fewer chemokines, they engage monocytes and stimulate the production of CCL2 by monocytes, thus promoting macrophage aggregation within the tumor microenvironment [59].

One study differently claimed that CCL2 is highly expressed in cervical cancer cells, and its expression level is related to the number of tumor-associated macrophages. The expression of CCL2 in cervical cancer cells causes the recruitment of macrophages, which supports the progression of the cervical tumor. Patients with cervical cancer who lack CCL2 expression have a better prognosis and longer overall survival [60].

In cervical cancer, serum CCL2 is elevated in cervical adenocarcinoma, according to the most recent study. When binding to its receptor CCR2 on the membrane of cervical cancer cells, the CCL2 generated from Schwann cells promoted cancer cell proliferation, migration, and invasion. Furthermore, CCL2 induced the epithelial-mesenchymal transition of cervical cancer cells, thereby promoting their metastasis and facilitating the perineural invasion of cervical cancer. Based on these results, the CCL2/CCR2 axis may serve as a potential marker for cervical cancer treatment [61].

### CCL2 and endometrial cancer

Previous studies have indicated that CCL2 plays a role in pathological processes in breast cancer, ovarian cancer, and cervical cancer. There is still a lack of understanding of CCL2’s role in endometrial cancer. In a study, the human endometrial stromal sarcoma cell line Mami constitutively produces CCL2 in vitro, and interleukin-1β, tumor necrosis factor α, and lipopolysaccharide-induced the expression of CCL2 in MaMi cells [62]. Furthermore, unfractionated heparin attenuated the expression of CCL2 in different endometrial adenocarcinoma cell lines (ECC-1, RL95–2, HEC-1A, KLE, and AN3CA) [63]. Interleukin-17 induced CCL2 mRNA levels in human endometrial cancer cell line HEC-1-B in particular by activating IκBα and extracellular signal-regulated kinase 1/2 [64]. Tamoxifen decreased CCL2 secretion and mRNA and protein levels in the human endometrial cancer cell line EFE-184. On the other hand, Buserelin had no effect on CCL2 expression [65]. Furthermore, Attar et al. demonstrated that the polymorphism of CCL2 and CCR2 is related to endometrial cancer [66].

Furthermore, numerous studies have shown that CCL2 regulates endometrial cancer progression through the recruitment of macrophages. Endometrial cancer cell proliferation decreased in vivo when the expression of activating transcription factor 4 (ATF4) was knocked down, as was the infiltration of M2 macrophages. Moreover, ATF4 regulated macrophage infiltration by mediating the expression of CCL2, which ultimately led to the growth of endometrial cancer [67]. Endometrial tumor growth was decreased by the serine/threonine kinase LKB1, and suppressing LKB1 resulted in elevated expression of CCL2, which leads to increased recruitment of macrophages. Furthermore, CCL2 inactivation also delayed the growth of endometrial cancer and improved survival [68].

## Summary and prospect

CCL2 stimulates the growth, metastasis, and invasion of gynecological tumors, regulates the formation of the tumor microenvironment and plays a key role in tumor progression (Table 1). The present research has confirmed that blocking the CCL2/CCR2 axis can prevent the growth of prostate and breast cancer cells. Consequently, CCL2/CCR2 axis can also be further studied as a new target for gynecological tumor therapy. Although the functions of CCL2 in gynecological tumors have been basically defined, the mechanisms of CCL2 in the gynecological tumor microenvironment are still unclear and need further exploration.

Interleukin-1β (IL-1β) is a key pro-inflammatory factor that belongs to the interleukin-1 (IL-1) family. IL-1β stimulates the inflammatory response by regulating the expression of downstream genes comprising CCL2. Tao et al. indicated that IL-1β stimulates cervical cancer cell proliferation, migration, and invasion by accelerating the production and release of CCL2 in an NF-κB phosphorylation-dependent manner [69]. Furthermore, blocking IL-1β can reduce the recruitment of monocytes mediated by CCL2, decrease the infiltrating macrophages in the tumor microenvironment, and prevent breast tumor growth [70]. Currently, our group found that both CCL2 and IL-1β promote the proliferation and metastasis of epithelial ovarian cancer cells. There is a correlation between CCL2 and IL-1β in the progression of ovarian cancer. Many additional cytokines and chemokines regulate the expression of CCL2 and co-mediate gynecological cancer in addition to IL-1. Understanding the role of CCL2 and its regulators in the microenvironment of gynecological tumors could lead to novel discoveries.

## Data Availability

Not applicable.

## References

[1] Balkwill FR (2012). The chemokine system and cancer. J Pathol.

[2] Gorbachev AV, Fairchild RL (2014). Regulation of chemokine expression in the tumor microenvironment. Crit Rev Immunol.

[3] Ling Z, Yang X, Chen X, Xia J, Cheng B, Tao X (2019). CCL2 promotes cell migration by inducing epithelial–mesenchymal transition in oral squamous cell carcinoma. J Oral Pathol Med.

[4] Zhuang H, Cao G, Kou C, Liu T (2018). CCL2/CCR2 axis induces hepatocellular carcinoma invasion and epithelial–mesenchymal transition in vitro through activation of the Hedgehog pathway. Oncol Rep.

[5] Zheng Y, Wang Z, Wei S, Liu Z, Chen G (2021). Epigenetic silencing of chemokine CCL2 represses macrophage infiltration to potentiate tumor development in small cell lung cancer. Cancer Lett.

[6] Yoshimura T (2018). The chemokine MCP-1 (CCL2) in the host interaction with cancer: a foe or ally?. Cell Mol Immunol.

[7] Gschwandtner M, Derler R, Midwood KS (2019). More than just attractive: how CCL2 influences myeloid cell behavior beyond chemotaxis. Front Immunol.

[8] Raghu H, Lepus CM, Wang Q, Wong HH, Lingampalli N, Oliviero F, Punzi L, Giori NJ, Goodman SB, Chu CR, Sokolove JB, Robinson WH (2017). CCL2/CCR2, but not CCL5/CCR5, mediates monocyte recruitment, inflammation and cartilage destruction in osteoarthritis. Ann Rheum Dis.

[9] Lehmann MH, Lehmann JM, Erfle V (2019). Nef-induced CCL2 expression contributes to HIV/SIV brain invasion and neuronal dysfunction. Front Immunol.

[10] Behfar S, Hassanshahi G, Nazari A, Khorramdelazad H (2018). A brief look at the role of monocyte chemoattractant protein-1 (CCL2) in the pathophysiology of psoriasis. Cytokine.

[11] O’Connor T, Borsig L, Heikenwalder M (2015). CCL2-CCR2 signaling in disease pathogenesis. Endocr Metab Immune Disord Drug Targets.

[12] Ishida Y, Kuninaka Y, Nosaka M, Furuta M, Kimura A, Taruya A, Yamamoto H, Shimada E, Akiyama M, Mukaida N, Kondo T (2019). CCL2-mediated reversal of impaired skin wound healing in diabetic mice by normalization of neovascularization and collagen accumulation. J Invest Dermatol.

[13] Whelan DS, Caplice NM, Clover AJP (2020). Mesenchymal stromal cell derived CCL2 is required for accelerated wound healing. Sci Rep.

[14] Lim SY, Yuzhalin AE, Gordon-Weeks AN, Muschel RJ (2016). Targeting the CCL2-CCR2 signaling axis in cancer metastasis. Oncotarget.

[15] Chen C, He W, Huang J, Wang B, Li H, Cai Q, Su F, Bi J, Liu H, Zhang B, Jiang N, Zhong G, Zhao Y, Dong W, Lin T (2018). LNMAT1 promotes lymphatic metastasis of bladder cancer via CCL2 dependent macrophage recruitment. Nat Commun.

[16] Yang J, Lv X, Chen J, Xie C, Xia W, Jiang C, Zeng T, Ye Y, Ke L, Yu Y, Liang H, Guan XY, Guo X, Xiang Y (2016). CCL2-CCR2 axis promotes metastasis of nasopharyngeal carcinoma by activating ERK1/2-MMP2/9 pathway. Oncotarget.

[17] Ding M, He SJ, Yang J (2019). MCP-1/CCL2 mediated by autocrine loop of PDGF-BB promotes invasion of lung cancer cell by recruitment of macrophages via CCL2-CCR2 axis. J Interferon Cytokine Res.

[18] Pausch TM, Aue E, Wirsik NM, Freire Valls A, Shen Y, Radhakrishnan P, Hackert T, Schneider M, Schmidt T (2020). Metastasis-associated fibroblasts promote angiogenesis in metastasized pancreatic cancer via the CXCL8 and the CCL2 axes. Sci Rep.

[19] Yang X, Lin Y, Shi Y, Li B, Liu W, Yin W, Dang Y, Chu Y, Fan J, He R (2016). FAP promotes immunosuppression by cancer-associated fibroblasts in the tumor microenvironment via STAT3-CCL2 signaling. Cancer Res.

[20] Zhang J, Yan Y, Cui X, Zhang J, Yang Y, Li H, Wu H, Li J, Wang L, Li M, Liu X, Wang J, Duan X (2017). CCL2 expression correlates with Snail expression and affects the prognosis of patients with gastric cancer. Pathol Res Pract.

[21] Wang Z, Xie H, Zhou L, Liu Z, Fu H, Zhu Y, Xu L, Xu J (2016). CCL2/CCR2 axis is associated with postoperative survival and recurrence of patients with non-metastatic clear-cell renal cell carcinoma. Oncotarget.

[22] Yumimoto K, Sugiyama S, Mimori K, Nakayama KI (2019). Potentials of C-C motif chemokine 2-C-C chemokine receptor type 2 blockers including propagermanium as anticancer agents. Cancer Sci.

[23] Zhang J, Sarkar S, Cua R, Zhou Y, Hader W, Yong VW (2012). A dialog between glioma and microglia that promotes tumor invasiveness through the CCL2/CCR2/interleukin-6 axis. Carcinogenesis.

[24] Shono K, Yamaguchi I, Mizobuchi Y, Kagusa H, Sumi A, Fujihara T, Nakajima K, Kitazato KT, Matsuzaki K, Saya H, Takagi Y (2020). Downregulation of the CCL2/CCR2 and CXCL10/CXCR3 axes contributes to antitumor effects in a mouse model of malignant glioma. Sci Rep.

[25] Han R, Gu S, Zhang Y, Luo A, Jing X, Zhao L, Zhao X, Zhang L (2018). Estrogen promotes progression of hormone-dependent breast cancer through CCL2-CCR2 axis by upregulation of Twist via PI3K/AKT/NF-κB signaling. Sci Rep.

[26] Yao M, Fang W, Smart C, Hu Q, Huang S, Alvarez N, Fields P, Cheng N (2019). CCR2 chemokine receptors enhance growth and cell-cycle progression of breast cancer cells through SRC and PKC activation. Mol Cancer Res.

[27] Macanas-Pirard P, Quezada T, Navarrete L, Broekhuizen R, Leisewitz A, Nervi B, Ramírez PA (2017). The CCL2/CCR2 axis affects transmigration and proliferation but not resistance to chemotherapy of acute myeloid leukemia cells. PLoS ONE.

[28] Li X, Yao W, Yuan Y, Chen P, Li B, Li J, Chu R, Song H, Xie D, Jiang X, Wang H (2017). Targeting of tumour-infiltrating macrophages via CCL2/CCR2 signalling as a therapeutic strategy against hepatocellular carcinoma. Gut.

[29] Yang Z, Li H, Wang W, Zhang J, Jia S, Wang J, Wei J, Lei D, Hu K, Yang X (2019). CCL2/CCR2 axis promotes the progression of salivary adenoid cystic carcinoma via recruiting and reprogramming the tumor-associated macrophages. Front Oncol.

[30] Yang H, Zhang Q, Xu M, Wang L, Chen X, Feng Y, Li Y, Zhang X, Cui W, Jia X (2020). CCL2-CCR2 axis recruits tumor associated macrophages to induce immune evasion through PD-1 signaling in esophageal carcinogenesis. Mol Cancer.

[31] Li D, Ji H, Niu X, Yin L, Wang Y, Gu Y, Wang J, Zhou X, Zhang H, Zhang Q (2020). Tumor-associated macrophages secrete CC-chemokine ligand 2 and induce tamoxifen resistance by activating PI3K/Akt/mTOR in breast cancer. Cancer Sci.

[32] Roblek M, Protsyuk D, Becker PF, Stefanescu C, Gorzelanny C, Garzon JFG, Knopfova L, Heikenwalder M, Luckow B, Schneider SW, Borsig L (2019). CCL2 is a vascular permeability factor inducing CCR2-dependent endothelial retraction during lung metastasis. Mol Cancer Res.

[33] Liang Y, Zhang H, Song X, Yang Q (2020). Metastatic heterogeneity of breast cancer: Molecular mechanism and potential therapeutic targets. Semin Cancer Biol.

[34] Lubowicka E, Przylipiak A, Zajkowska M, Piskór BM, Malinowski P, Fiedorowicz W, Ławicki S (2018). Plasma chemokine CCL2 and its receptor CCR2 concentrations as diagnostic biomarkers for breast cancer patients. Biomed Res Int.

[35] Heiskala M, Leidenius M, Joensuu K, Heikkilä P (2019). High expression of CCL2 in tumor cells and abundant infiltration with CD14 positive macrophages predict early relapse in breast cancer. Virchows Arch.

[36] Szekely B, Bossuyt V, Li X, Wali VB, Patwardhan GA, Frederick C, Silber A, Park T, Harigopal M, Pelekanou V, Zhang M, Yan Q, Rimm DL, Bianchini G, Hatzis C, Pusztai L (2018). Immunological differences between primary and metastatic breast cancer. Ann Oncol.

[37] Fang WB, Jokar I, Zou A, Lambert D, Dendukuri P, Cheng N (2012). CCL2/CCR2 chemokine signaling coordinates survival and motility of breast cancer cells through Smad3 protein- and p42/44 mitogen-activated protein kinase (MAPK)-dependent mechanisms. J Biol Chem.

[38] Xiong W, Tan J, Guo Y, Chen S, Fan L, Li Y (2020). Notch3 knockout suppresses mouse mammary gland development and inhibits the proliferation of 4T1 murine mammary carcinoma cells via CCL2/CCR4 axis. Front Cell Dev Biol.

[39] Linde N, Casanova-Acebes M, Sosa MS, Mortha A, Rahman A, Farias E, Harper K, Tardio E, Reyes Torres I, Jones J, Condeelis J, Merad M, Aguirre-Ghiso JA (2018). Macrophages orchestrate breast cancer early dissemination and metastasis. Nat Commun.

[40] Qian BZ, Li J, Zhang H, Kitamura T, Zhang J, Campion LR, Kaiser EA, Snyder LA, Pollard JW (2011). CCL2 recruits inflammatory monocytes to facilitate breast-tumour metastasis. Nature.

[41] Kitamura T, Qian BZ, Soong D, Cassetta L, Noy R, Sugano G, Kato Y, Li J, Pollard JW (2015). CCL2-induced chemokine cascade promotes breast cancer metastasis by enhancing retention of metastasis-associated macrophages. J Exp Med.

[42] Arendt LM, McCready J, Keller PJ, Baker DD, Naber SP, Seewaldt V, Kuperwasser C (2013). Obesity promotes breast cancer by CCL2-mediated macrophage recruitment and angiogenesis. Cancer Res.

[43] Fang WB, Yao M, Brummer G, Acevedo D, Alhakamy N, Berkland C, Cheng N (2016). Targeted gene silencing of CCL2 inhibits triple negative breast cancer progression by blocking cancer stem cell renewal and M2 macrophage recruitment. Oncotarget.

[44] Brummer G, Fang W, Smart C, Zinda B, Alissa N, Berkland C, Miller D, Cheng N (2020). CCR2 signaling in breast carcinoma cells promotes tumor growth and invasion by promoting CCL2 and suppressing CD154 effects on the angiogenic and immune microenvironments. Oncogene.

[45] Yao M, Smart C, Hu Q, Cheng N (2017). Continuous delivery of neutralizing antibodies elevate CCL2 levels in mice bearing MCF10CA1d breast tumor xenografts. Transl Oncol.

[46] Stewart C, Ralyea C, Lockwood S (2019). Ovarian cancer: an integrated review. Semin Oncol Nurs.

[47] Hefler L, Tempfer C, Heinze G, Mayerhofer K, Breitenecker G, Leodolter S, Reinthaller A, Kainz C (1999). Monocyte chemoattractant protein-1 serum levels in ovarian cancer patients. Br J Cancer.

[48] Geller MA, Bui-Nguyen TM, Rogers LM, Ramakrishnan S (2010). Chemotherapy induces macrophage chemoattractant protein-1 production in ovarian cancer. Int J Gynecol Cancer.

[49] Furukawa S, Soeda S, Kiko Y, Suzuki O, Hashimoto Y, Watanabe T, Nishiyama H, Tasaki K, Hojo H, Abe M, Fujimori K (2013). MCP-1 promotes invasion and adhesion of human ovarian cancer cells. Anticancer Res.

[50] Liu W, Wang L, Zhang J, Qiao L, Liu Y, Yang X, Zhang J, Zheng W, Ma Z (2021). Purification of recombinant human chemokine CCL2 in E. coli and its function in ovarian cancer. 3 Biotech.

[51] Deng W, Gu X, Lu Y, Gu C, Zheng Y, Zhang Z, Chen L, Yao Z, Li LY (2012). Down-modulation of TNFSF15 in ovarian cancer by VEGF and MCP-1 is a pre-requisite for tumor neovascularization. Angiogenesis.

[52] Pasquier J, Gosset M, Geyl C, Hoarau-Véchot J, Chevrot A, Pocard M, Mirshahi M, Lis R, Rafii A, Touboul C (2018). CCL2/CCL5 secreted by the stroma induce IL-6/PYK2 dependent chemoresistance in ovarian cancer. Mol Cancer.

[53] Yasui H, Kajiyama H, Tamauchi S, Suzuki S, Peng Y, Yoshikawa N, Sugiyama M, Nakamura K, Kikkawa F (2020). CCL2 secreted from cancer-associated mesothelial cells promotes peritoneal metastasis of ovarian cancer cells through the P38-MAPK pathway. Clin Exp Metastasis.

[54] Sun C, Li X, Guo E, Li N, Zhou B, Lu H, Huang J, Xia M, Shan W, Wang B, Li K, Weng D, Xu X, Gao Q, Wang S, Hu J, Lu Y, Mills GB, Chen G (2020). MCP-1/CCR-2 axis in adipocytes and cancer cell respectively facilitates ovarian cancer peritoneal metastasis. Oncogene.

[55] Arbyn M, Weiderpass E, Bruni L, de Sanjosé S, Saraiya M, Ferlay J, Bray F (2020). Estimates of incidence and mortality of cervical cancer in 2018: a worldwide analysis. Lancet Glob Health.

[56] Adiga D, Eswaran S, Pandey D, Sharan K, Kabekkodu SP (2021). Molecular landscape of recurrent cervical cancer. Crit Rev Oncol Hematol.

[57] Kleine-Lowinski K, Gillitzer R, Kühne-Heid R, Rösl F (1999). Monocyte-chemo-attractant-protein-1 (MCP-1)-gene expression in cervical intra-epithelial neoplasias and cervical carcinomas. Int J Cancer.

[58] Riethdorf S, Riethdorf L, Richter N, Löning T (1998). Expression of the MCP-1 gene and the HPV 16 E6/E7 oncogenes in squamous cell carcinomas of the cervix uteri and metastases. Pathobiology.

[59] Pahne-Zeppenfeld J, Schröer N, Walch-Rückheim B, Oldak M, Gorter A, Hegde S, Smola S (2014). Cervical cancer cell-derived interleukin-6 impairs CCR7-dependent migration of MMP-9-expressing dendritic cells. Int J Cancer.

[60] Zijlmans HJ, Fleuren GJ, Baelde HJ, Eilers PH, Kenter GG, Gorter A (2006). The absence of CCL2 expression in cervical carcinoma is associated with increased survival and loss of heterozygosity at 17q11.2. J Pathol.

[61] Huang T, Fan Q, Wang Y, Cui Y, Wang Z, Yang L, Sun X, Wang Y (2020). Schwann cell-derived CCL2 promotes the perineural invasion of cervical cancer. Front Oncol.

[62] Nasu K, Matsui N, Narahara H, Tanaka Y, Takai N, Miyakawa I, Higuchi Y (1998). MaMi, a human endometrial stromal sarcoma cell line that constitutively produces interleukin-6, interleukin-8, and monocyte chemoattractant protein-1. Arch Pathol Lab Med.

[63] Doster A, Schwarzig U, Zygmunt M, Rom J, Schütz F, Fluhr H (2016). Unfractionated heparin selectively modulates the expression of CXCL8, CCL2 and CCL5 in endometrial carcinoma cells. Anticancer Res.

[64] Lai T, Wang K, Hou Q, Zhang J, Yuan J, Yuan L, You Z, Xi M (2011). Interleukin 17 induces up-regulation of chemokine and cytokine expression via activation of the nuclear factor κB and extracellular signal-regulated kinase 1/2 pathways in gynecologic cancer cell lines. Int J Gynecol Cancer.

[65] Wang L, Zheng W, Zhang S, Chen X, Hornung D (2006). Expression of monocyte chemotactic protein-1 in human endometrial cancer cells and the effect of treatment with tamoxifen or buserelin. J Int Med Res.

[66] Attar R, Agachan B, Kuran SB, Cacina C, Sozen S, Yurdum LM, Attar E, Isbir T (2010). Association of CCL2 and CCR2 gene variants with endometrial cancer in Turkish women. In Vivo.

[67] Liu B, Chen P, Xi D, Zhu H, Gao Y (2017). ATF4 regulates CCL2 expression to promote endometrial cancer growth by controlling macrophage infiltration. Exp Cell Res.

[68] Peña CG, Nakada Y, Saatcioglu HD, Aloisio GM, Cuevas I, Zhang S, Miller DS, Lea JS, Wong K, DeBerardinis RJ, Amelio AL, Brekken RA, Castrillon DH (2015). LKB1 loss promotes endometrial cancer progression via CCL2-dependent macrophage recruitment. J Clin Invest.

[69] Tao L, Liu S, Xiong J, Yang H, Wu Y, Xu A, Gong Y (2021). IL-1β promotes cervical cancer through activating NF-κB/CCL-2. Int J Clin Exp Pathol.

[70] Kaplanov I, Carmi Y, Kornetsky R, Shemesh A, Shurin GV, Shurin MR, Dinarello CA, Voronov E, Apte RN (2019). Blocking IL-1β reverses the immunosuppression in mouse breast cancer and synergizes with anti-PD-1 for tumor abrogation. Proc Natl Acad Sci USA.

